# Associations of Circulating Lymphocyte Subpopulations with Type 2 Diabetes: Cross-Sectional Results from the Multi-Ethnic Study of Atherosclerosis (MESA)

**DOI:** 10.1371/journal.pone.0139962

**Published:** 2015-10-12

**Authors:** Nels C. Olson, Margaret F. Doyle, Ian H. de Boer, Sally A. Huber, Nancy Swords Jenny, Richard A. Kronmal, Bruce M. Psaty, Russell P. Tracy

**Affiliations:** 1 Department of Pathology and Laboratory Medicine, University of Vermont College of Medicine, Burlington, Vermont, United States of America; 2 Division of Nephrology, Department of Medicine, University of Washington, Seattle, Washington, United States of America; 3 Kidney Research Institute, University of Washington, Seattle, Washington, United States of America; 4 Collaborative Health Studies Coordinating Center, Department of Biostatistics, University of Washington, Seattle, Washington, United States of America; 5 Departments of Medicine, Epidemiology, Health Services, and Cardiovascular Health Research Unit, University of Washington, Seattle, Washington, United States of America; 6 Group Health Research Institute, Group Health Cooperative, Seattle, Washington, United States of America; 7 Department of Biochemistry, University of Vermont College of Medicine, Burlington, Vermont, United States of America; University of Catanzaro Magna Graecia, ITALY

## Abstract

**Objective:**

Distinct lymphocyte subpopulations have been implicated in the regulation of glucose homeostasis and obesity-associated inflammation in mouse models of insulin resistance. Information on the relationships of lymphocyte subpopulations with type 2 diabetes remain limited in human population-based cohort studies.

**Methods:**

Circulating levels of innate (γδ T, natural killer (NK)) and adaptive immune (CD4^+^ naive, CD4^+^ memory, Th1, and Th2) lymphocyte subpopulations were measured by flow cytometry in the peripheral blood of 929 free-living participants of the Multi-Ethnic Study of Atherosclerosis (MESA). Cross-sectional relationships of lymphocyte subpopulations with type 2 diabetes (*n* = 154) and fasting glucose and insulin concentrations were evaluated by generalized linear models.

**Results:**

Each standard deviation (SD) higher CD4^+^ memory cells was associated with a 21% higher odds of type 2 diabetes (95% CI: 1–47%) and each SD higher naive cells was associated with a 22% lower odds (95% CI: 4–36%) (adjusted for age, gender, race/ethnicity, and BMI). Among participants not using diabetes medication, higher memory and lower naive CD4^+^ cells were associated with higher fasting glucose concentrations (p<0.05, adjusted for age, sex, and race/ethnicity). There were no associations of γδ T, NK, Th1, or Th2 cells with type 2 diabetes, glucose, or insulin.

**Conclusions:**

A higher degree of chronic adaptive immune activation, reflected by higher memory and lower naive CD4^+^ cells, was positively associated with type 2 diabetes. These results are consistent with a role of chronic immune activation and exhaustion augmenting chronic inflammatory diseases, and support the importance of prospective studies evaluating adaptive immune activation and type 2 diabetes.

## Introduction

Chronic low-grade inflammation is an important component of insulin resistance (IR) and the progression to type 2 diabetes mellitus [1]. Elevated circulating biomarkers of inflammation are a characteristic feature of type 2 diabetes [2]. C-reactive protein (CRP), interleukin-6 (IL-6), plasminogen activator inhibitor-1 (PAI-1), and IL-1β predict the future occurrence of disease [3–5].

Activation of the innate immune system is a major component of chronic inflammation. Proinflammatory cytokines, such as tumor necrosis factor alpha (TNF-α) and IL-6, generated during the innate response, can inhibit insulin signaling through the phosphorylation of insulin receptor substrate 1 (IRS-1) [6, 7], serving to link inflammation with IR and type 2 diabetes [8]. Adipocytes and macrophages are thought to be primary sources of proinflammatory mediators in type 2 diabetes and are considered centrally important in disease pathogenesis [9, 10].

Emerging evidence has suggested involvement of distinct lymphocyte subpopulations during obesity-associated inflammation, IR, and type 2 diabetes [11, 12]. Lymphocytes include both innate (γδ T, natural killer (NK)) and adaptive immune (CD4^+^ T-lymphocyte, CD8^+^ T-lymphocyte, B-lymphocyte) subpopulations; CD4^+^ T-lymphocytes are further subdivided as T helper (Th) type 1 (Th1), Th2, Th17, Th22, and T regulatory cells (Treg).

The proportions of lymphocyte subpopulations localized to adipose tissue is altered between lean and high-fat diet-fed mice. Subpopulations of proinflammatory γδ T, Th1, and CD8^+^ T cells were increased in response to a high-fat diet [12, 13], along with a concomitant reduction of anti-inflammatory NK, Th2, and Treg cells [12, 14]. Secretion of the proinflammatory cytokines IFN-γ and IL-17 by Th1 and Th17 cells were implicated in the promotion of insulin resistance [12, 15, 16]. Lymphocyte-deficient mice fed a high-fat diet, however, developed a diabetic phenotype. Repletion of CD4^+^ T cells reversed weight gain, insulin resistance, and glucose intolerance primarily through Th2-dependent regulation of glucose homeostasis [12]. These findings suggested both inflammatory and regulatory roles of distinct lymphocyte subpopulations. Although the mechanisms responsible for lymphocyte-associated metabolic regulation remain unclear, cytokine-mediated induction of proinflammatory M1 and anti-inflammatory M2 macrophage polarization in adipose tissue is considered a key factor [12, 17].

Evaluation of lymphocyte subpopulations in the pathogenesis of type 2 diabetes in humans remains limited. Altered lymphocyte profiles were reported in obesity [12, 18–21], a major risk factor for type 2 diabetes. A small number of clinical studies described higher Th1, Th17, and Th22 subpopulations and lower Tregs in adipose tissue biopsies or peripheral blood of those with prediabetes or type 2 diabetes [21–27]. Impaired NK cell receptor activation and enhanced methylation profiles were also reported in type 2 diabetes patients [28, 29].

The relationships of distinct lymphocyte subpopulations with type 2 diabetes has not been evaluated in free-living population-based cohorts. Although type 2 diabetes is not considered a conventional autoimmune disease *per se*, the current findings in animal models and clinical studies suggest the importance of examining the relationships of lymphocyte subpopulations with IR and type 2 diabetes in population-based studies [30].

We evaluated the cross-sectional relationships of circulating NK cells, γδ T-cells, CD4^+^ naive T-cells, CD4^+^ memory T-cells, Th1, and Th2 cells with prevalent type 2 diabetes in 929 free-living participants of the Multi-Ethnic Study of Atherosclerosis (MESA). We had three main hypotheses: first, high levels of proinflammatory lymphocyte subpopulations, including γδ T and Th1 cells, would be positively associated with prevalent type 2 diabetes; second, that high levels of anti-inflammatory lymphocyte subpopulations including NK and Th2 cells, would be inversely associated with type 2 diabetes; and, third, a high degree of chronic adaptive immune activation, as characterized by higher memory and lower naive CD4^+^ T cells, would be positively associated with type 2 diabetes.

## Materials and Methods

### Study population and ethics statement

MESA is a multi-ethnic, population-based longitudinal epidemiological study initiated in 2000 to investigate subclinical cardiovascular disease (CVD) [31]. MESA includes 6,814 European-American, African-American, Hispanic-American, and Chinese-American men and women recruited from six field centers (Baltimore MD, Chicago IL, Los Angeles CA, St. Paul MN, New York NY, and Forsyth County NC), aged 45–84 years, and free of known clinical CVD during the baseline exam (exam 1) in 2000–2002. Autoimmune or infectious diseases were not considered exclusionary for enrollment in MESA. All MESA participants gave written informed consent for participation in the study. The institutional review boards of the six field centers: Johns Hopkins University School of Medicine Institutional Review Boards; Northwestern University Institutional Review Board; University of California, Los Angeles Institutional Review Board; The University of Minnesota Institutional Review Board; Columbia University Medical Center Institutional Review Board; and, Wake Forest University Health Sciences Institutional Review Board approved the study protocol.

As part of MESA exam 1, a random subset of 1000 participants were selected for specialized biomarker analysis. During MESA exam 4 (2005–2007), the subset selected for specialized biomarker analysis, plus replacements from the rest of the cohort for those who died or dropped out of the study, were included in the MESA-Inflammation ancillary study for analysis of cellular phenotypes. The present study included participants with data for at least one of the lymphocyte phenotypes being evaluated (*n* = 929).

During exam 4, whole blood samples were collected at the six field centers and sent overnight in specialized containers to the Laboratory for Clinical Biochemistry Research at the University of Vermont [32]. Exam 5 occurred in 2010–2012, at which time additional outcome measurements (insulin and hemoglobin A1c (HbA1c)) were obtained. Analyses in this study used data from MESA exam 4 to investigate cross-sectional associations. When data were not available from exam 4, data from exam 5 were used in the analyses. Both the parent MESA study and MESA-Inflammation ancillary study received IRB approval and written informed consent was obtained from all study participants.

### Cellular Phenotype Assays

A full description of the MESA-Inflammation cellular phenotyping design, methods, and quality assurance / quality control program have been published [32, 33]. All cellular phenotypes were measured during MESA exam 4. Cells were analyzed by flow cytometry using an LSR II flow cytometer (BD Biosciences, San Jose, CA). Lymphocytes (*n* ≥30,000) were gated based on their forward and side scatter. Single color controls were used to set machine compensation, and background staining was determined using isotype-matched controls for each assay. Data were analyzed using WinList 6.0 (Verity Software House; Topsham, ME). Typical flow cytometry gating strategies and additional quality control details are found in the Supporting Information (S1 File; S1, S3 and S4 Figs).

γδ T cells and NK cells were measured from 100 μL of whole blood incubated with FITC anti-CD3, PE anti-γδ T-cell receptor (TCR), Alexa-647 anti-CD16 (BD Biosciences), and PECy5.5 anti-CD56 (Invitrogen, Carlsbad, CA) according to the manufacturer’s recommendations. FITC-conjugated mouse IgG1 κ, PE-conjugated mouse IgG1 κ, Alexa-647 conjugated mouse IgG1 κ (BD Biosciences), and PE-Cy5.5-conjugated mouse IgG2a (Invitrogen) were analyzed as isotype matched controls. γδ T cells were defined as CD3^+^γδTCR^+^ and were expressed as a percentage of lymphocytes; NK cells were defined as CD3^-^CD56^+^CD16^+^ and expressed as percentage of lymphocytes. The distributions of γδ T and NK cells in participants of MESA-Inflammation are shown in S2 Fig.

CD4^+^ memory and naive T-cells were measured in whole blood (100 μL) as described [33], incubated with APC-Cy7 anti-CD4, FITC anti-CD45RO, and PE-Cy5.5 anti-CD45RA (Invitrogen). Memory and naive cells were expressed as the percentage of CD4^+^ lymphocytes that were CD45RO^+^ (%memory: CD4^+^CD45RO^+^) or CD45RA^+^ (%naive: CD4^+^CD45RA^+^).

Th1 and Th2 cells were measured from peripheral blood mononuclear cells (PBMCs) as described [32]. PBMCs were stimulated with phorbol 12-myristate 13-acetate (PMA; 40 ng/mL), ionomycin (1 μg/mL), and Brefeldin A (10 μg/mL) (Sigma-Aldrich, St. Louis, MO) for 3 hours at 37°C and labeled with PE-Cy5 anti-CD4 (BD Biosciences). Cells were fixed with 2% paraformaldehyde, permeabilized with 0.1% saponin, and incubated with FITC anti-IFN-γ and PE anti-IL-4 (Sigma-Aldrich, St. Louis, MO). Unstimulated PBMCs that underwent the same protocol without the addition of activators were used as negative controls. Th1 and Th2 cells were expressed as the percentage of CD4^+^ lymphocytes that were IFN-γ^+^ (%Th1: CD4^+^IFN-γ^+^) or IL-4^+^ (%Th2: CD4^+^IL-4^+^).

### Laboratory Measurements

Fasting blood glucose measurements were performed at the Collaborative Studies Clinical Laboratory at University of Minnesota Medical Center, Fairview (Minneapolis, MN). Serum glucose was measured at MESA exam 4 using a Vitros analyzer (Johnson & Johnson Clinical Diagnostics, Inc., Rochester, NY); coefficient of variation (CV) 1.1%. HbA1c was measured at MESA exam 5 by high performance liquid chromatography (HPLC) using a Tosoh A1c 2.2 Plus HPLC Glycohemoglobin Analyzer (Tosoh Medics, Inc., San Francisco, CA); CV 1.4–1.9%. Fasting serum insulin was measured during exam 5 by an automated sandwich immunoassay on a Roche Elecsys 2010 Analyzer (Roche Diagnostics, Indianapolis, IN); CV 3.3–4.2%. Cytomegalovirus (CMV) and *H*. *pylori* serologies were measured during exam 1 as described [34].

### Definitions

BMI was defined as weight in kilograms divided by height in meters squared (kg/m^2^). Waist circumference measurements were in centimeters. Smoking was defined as never, former (no cigarettes within the past 30 days), or current. Medication use was determined by medication inventory. Type 2 diabetes status was from MESA exam 4, classified by the 2003 American Diabetes Association (ADA) criteria [35]. Participants were considered to have diabetes if they had a fasting blood glucose ≥126 mg/dL or used hypoglycemic medication. Participants were considered to have impaired fasting glucose (IFG) if they did not have diabetes by the preceding criteria and if their fasting blood glucose was between 100–125 mg/dL.

### Statistical Analyses

All statistical analyses were conducted using the Statistical Analysis System (SAS, version 9.3; SAS Institute, Inc., Cary, NC). NK and γδ T cells were analyzed as proportions of lymphocytes (%NK, %γδ T); CD4^+^ T-lymphocyte subpopulations were analyzed as a proportion of CD4^+^ cells (%naive, %memory, %Th1, %Th2). %γδ T had a highly skewed distribution (Panel B in S2 Fig) and was natural logarithm (ln) transformed. Descriptive data were stratified by type 2 diabetes status and differences were assessed using *t*-tests and analysis of variance (ANOVA) for continuous variables and χ^2^ statistics for categorical variables. Comparisons of lymphocyte subpopulation means by glycemic status were evaluated using ANOVA, adjusting for age, gender, and race/ethnicity, with a Tukey test *post hoc* to maintain the overall type I error rate at 5% for multiple comparisons.

Logistic regression models were used to calculate odds ratios (OR) of lymphocyte subpopulations with prevalent type 2 diabetes. Cell subpopulations were analyzed as quartiles of the distribution or per standard deviation (SD) increment higher in separate models with type 2 diabetes as the outcome. The %Th1:%Th2 ratio was also analyzed as an independent variable. Models included age, gender, and race/ethnicity as covariates in one model (demographic-adjusted), and these variables plus BMI, and separately waist circumference, in additional models.

With 152 participants with type 2 diabetes, 765 participants without diabetes, and an alpha level of 0.05, we had 80% power to detect a 0.25 SD difference in %Th1 cells between those with and without diabetes. This translated to a 2% absolute difference in %Th1 cell means (overall mean %Th1 = 15.9%). For %Th2 cells, we had 80% power to detect a 0.25 SD difference which translated to a 0.17% absolute difference in means (overall mean %Th2 = 0.83%). For ORs, we had 80% power to detect an OR of 1.74 for a lymphocyte subpopulation with a 25% prevalence (i.e., comparing the upper to the lower quartile) with an alpha level of 0.05.

As CMV and *H*. *pylori* titers are known correlates of %naive and %memory phenotypes in MESA [33], we planned *a*. *priori* to include additional adjustment for serological titers of these pathogens in analyses of %naive and %memory with type 2 diabetes. Covariates such as age, BMI, and waist circumference were used from the same exam as the outcome variable. Covariates in models of type 2 diabetes and glucose were from exam 4; for models of HbA1c and insulin, covariates and type 2 diabetes status were from exam 5.

The distributions of glucose and insulin were highly skewed, so natural logarithm transformations were used. Relationships with cell subpopulations were analyzed by linear regression with glucose, HbA1c, or insulin modeled as continuous dependent outcomes. Subjects treated with hypoglycemic medication were excluded from analyses of glucose, HbA1c, and insulin.

For the lymphocyte subpopulations significantly associated with type 2 diabetes, a sensitivity analysis was performed by stratifying on hypoglycemic medication status. All analyses were repeated with additional adjustment for hypertension or statin use, and by excluding all participants using steroids or with prevalent clinical CVD at MESA exam 4.

## Results

### Associations of lymphocyte subpopulations with type 2 diabetes

Among the MESA-Inflammation participants, 21% of the study population had impaired fasting glucose (IFG) (*n* = 196) and 17% had type 2 diabetes (*n* = 154); 13% of those with type 2 diabetes were untreated (*n* = 20) (Table 1).

**Table 1:** Characteristics of the MESA-Inflammation study population by type 2 diabetes status.

	MESA-Inflammation		
Variable	Non-Diabetic (*n* = 775)	T2D (*n* = 154)	P-value
Age, years (mean, SD)	65.4 (10.0)	66.9 (9.5)	0.09
Men (n, %)	363 (47)	84 (55)	0.08
Ethnicity (n, %)			0.002
European-American	355 (46)	49 (32)	
African-American	198 (25)	38 (25)	
Hispanic-American	146 (19)	45 (29)	
Chinese-American	76 (10)	22 (14)	
Waist Circumference, cm (mean, SD)	97.7 (13.8)	109.1 (16.4)	<0.0001
BMI, kg/m2 (mean, SD)	28.0 (5.1)	32.0 (6.7)	<0.0001
Smoking Status (n, %)			0.78
Never	342 (44)	67 (44)	
Former	356 (46)	74 (48)	
Current	73 (10)	12 (8)	
Hypertension Status (n, %)			<0.0001
Normal	412 (54)	43 (28)	
Hypertensive (takes medications)	349 (46)	108 (72)	
Glucose, mmol/L (median, 25^th^, 75^th^)	5.2 (4.8, 5.6)	7.1 (6.0, 8.5)	<0.0001
Impaired Fasting Glucose (n, %)	196 (21)	n/a	n/a
HbA1c*, % (median, 25^th^, 75^th^)	5.6 (5.5, 5.9)	6.7 (6.2, 7.4)	<0.0001
Insulin*, pmol/L (median, 25^th^, 75^th^)	329.9 (208.4, 507.0)	427.1 (291.7, 701.4)	0.0001
Takes Diabetes Medication (n, %)	n/a	134 (87)	n/a
Clinical CVD (*n*, %) **	40 (5)	13 (8)	0.11
Lymphocyte Indices			
%Natural Killer (mean, SD)	8.3 (5.2)	8.8 (5.6)	0.26
%γδ T (median, 25^th^, 75^th^)	1.7 (0.97, 3.0)	1.6 (0.99, 2.5)	0.87
%CD4 Naive (mean, SD)	29.0 (14.3)	24.6 (13.3)	0.0004
%CD4 Memory (mean, SD)	53.3 (15.0)	57.2 (15.5)	0.003
%Th1 (mean, SD)	15.8 (8.2)	16.6 (8.5)	0.28
%Th2 (mean, SD)	0.83 (0.70)	0.86 (0.65)	0.66

Data are from MESA exam 4 (2005–2007) unless otherwise noted.

*: Data are from MESA exam 5 (2010–2012).

**: Includes all participants with incident cardiovascular events (myocardial infarction, resuscitated cardiac arrest, definite or probable angina, and stroke) from baseline through the start of MESA exam 4.

Compared to those without diabetes, participants with type 2 diabetes had modestly higher mean (SD) values of %memory [57.2% (15.0%) vs 54.0% (16.8%)] (p = 0.02, adjusted for age, gender, and race/ethnicity) and lower mean (SD) values of %naive [25.1% (13.9%) vs 28.6% (15.7%)] (p = 0.005). There were no significant differences among the mean values of %NK, %γδ T, %Th1 or %Th2 by diabetes status (Table 1).

There were no significant differences in the mean levels of any lymphocyte subpopulation for participants with normal fasting glucose (NFG) compared to those with IFG. For example, the mean (SD) value of %memory was 53.8% (16.6%) for those with NFG and 54.5% (15.4%) for those with IFG (p = 0.57, adjusted for age, gender, and race/ethnicity). For %naive the mean value was 29.1% (15.6%) for participants with NFG and 27.1% (14.3%) for those with IFG (p = 0.09).

In demographic-adjusted logistic regression models, higher %memory was positively associated with prevalent type 2 diabetes. The odds ratio (OR) (95% confidence interval (CI)) per SD higher %memory was 1.25 (1.04, 1.50) (p = 0.02) (Table 2). In separate models, higher %naive was inversely associated with type 2 diabetes. The OR (95% CI) per SD higher %naive was 0.75 (0.62, 0.91) (p = 0.004). Adding further adjustment for BMI partially attenuated the results, but the associations remained statistically significant (Table 2).

**Table 2:** Associations of lymphocyte subpopulations with prevalent type 2 diabetes.

Lymphocyte subpopulation	Total *n*	Type 2 diabetes *n*	Model 1 OR (95% CI)	Model 2 OR (95% CI)
**Natural Killer**				
0.08–4.7%	223	31	1 (ref)	1 (ref)
4.7–7.1%	219	38	1.39 (0.82, 2.34)	1.26 (0.72, 2.19)
7.1–10.7%	227	43	1.42 (0.85, 2.38)	1.34 (0.78, 2.30)
10.7–37.3%	222	37	1.12 (0.65, 1.92)	1.01 (0.57, 1.78)
Per SD (5%)	891	149	1.06 (0.88, 1.26)	1.02 (0.84, 1.23)
p-value			0.56	0.85
**γδ T**				
0.06–0.97%	228	37	1 (ref)	1 (ref)
0.97–1.64%	222	44	1.28 (0.79, 2.10)	1.34 (0.80, 2.26)
1.64–2.86%	226	43	1.21 (0.74, 1.97)	1.18 (0.70, 1.99)
2.86–34.24%	225	27	0.65 (0.38, 1.12)	0.71 (0.40, 1.25)
Per SD (0.86)	901	151	0.95 (0.79, 1.13)	0.99 (0.82, 1.19)
p-value			0.56	0.91
**CD4** ^**+**^ **Naive**				
2.6–17.5%	228	54	1 (ref)	1 (ref)
17.5–26.8%	229	35	0.62 (0.38, 0.99)	0.61 (0.37, 1.02)
26.8–37.9%	227	36	0.66 (0.41, 1.06)	0.67 (0.40, 1.11)
37.9–78.0%	228	26	0.48 (0.28, 0.81)	0.55 (0.32, 0.96)
Per SD (14%)	912	151	0.75 (0.62, 0.91)	0.78 (0.64, 0.96)
p-value			0.004	0.02
**CD4** ^**+**^ **Memory**				
15.9–42.4%	231	29	1 (ref)	1 (ref)
42.4–54.0%	227	32	1.11 (0.65, 1.93)	1.16 (0.65, 2.07)
54.0–64.9%	229	38	1.35 (0.79, 2.29)	1.23 (0.70, 2.15)
64.9–92.6%	230	52	1.82 (1.10, 3.03)	1.74 (1.02, 2.99)
Per SD (15%)	917	151	1.25 (1.04, 1.50)	1.21 (1.01, 1.47)
p-value			0.02	0.04
**Th1**				
1.5–9.8%	230	38	1 (ref)	1 (ref)
9.8–14.5%	229	32	0.78 (0.46, 1.30)	0.65 (0.37, 1.13)
14.5–20.0%	226	36	0.87 (0.52, 1.44)	0.82 (0.48, 1.39)
20.0–60.0%	232	46	1.14 (0.70, 1.86)	1.06 (0.63, 1.77)
Per SD (8%)	917	152	1.05 (0.89, 1.25)	1.03 (0.86, 1.24)
p-value			0.56	0.75
**Th2**				
0.03–0.35%	224	37	1 (ref)	1 (ref)
0.35–0.63%	233	32	0.70 (0.41, 1.18)	0.58 (0.34, 1.01)
0.63–1.08%	227	42	1.00 (0.61, 1.65)	0.85 (0.50, 1.44)
1.08–4.5%	229	41	0.90 (0.54, 1.49)	0.74 (0.43, 1.26)
Per SD (0.7%)	913	152	0.98 (0.82, 1.17)	0.93 (0.77, 1.12)
p-value			0.79	0.44

P-values are from continuous models. Model 1: Age, gender, race/ethnicity. Model 2: Age, gender, race/ethnicity, and BMI.

Analyses adjusting for waist circumference in place of BMI yielded similar results, with an attenuation of the p-value for %memory. With waist circumference included in the models, the OR (95% CI) was 1.20 (0.99, 1.46) (p = 0.06) for %memory and 0.78 (0.64, 0.96) for %naive (p = 0.02). There were no associations of %NK, %γδ T, %Th1, %Th2, or the %Th1:%Th2 ratio with type 2 diabetes (Table 2).

In analyses that added further adjustment for CMV and *H*. *pylori* titers, the association of %memory, but not %naive, with type 2 diabetes was attenuated. The type 2 diabetes OR (95% CI) was 1.16 (0.92, 1.47) (p = 0.20) for %memory and 0.75 (0.59, 0.97) (p = 0.02) for %naive (adjusted for age, sex, race/ethnicity, BMI, and CMV and *H*. *pylori* titers).

In analyses of type 2 diabetes that stratified on treatment with hypoglycemic medication, the OR (95% CI) per SD higher %memory was 1.50 (0.94, 2.38) (p = 0.09) for non-pharmacologically treated participants (*n* = 20) and 1.22 (1.01, 1.48) (p = 0.04) for pharmacologically treated participants (*n* = 131). For %naive the ORs (95% CI) were 0.54 (0.31, 0.93) (p = 0.03) and 0.78 (0.64, 0.96) (p = 0.02), respectively. Adjusting further for BMI, or waist circumference, partially attenuated the results.

### Associations of lymphocyte subpopulations with glucose, insulin, and HbA1c

Among participants not using diabetes medication (*n* = 795; 775 without type 2 diabetes and 20 with non-pharmacologically treated diabetes), excluding those with pharmacological treatment for type 2 diabetes (*n* = 134), %memory and %naive were significantly associated with fasting glucose concentrations (Table 3). Each SD higher %memory was associated with 0.01 higher lnglucose (5% of a SD in lnglucose), and each SD higher %naive was associated with 0.02 lower lnglucose (7% of a SD in lnglucose). Associations were attenuated after additional adjustment for BMI, but remained statistically significant for %naive (Table 3). Results were essentially unaltered in analyses with adjustment for waist circumference in place of BMI (%naive β = -0.01 (SE = 0.005); p = 0.009).

**Table 3:** Associations of lymphocyte subpopulations with fasting glucose, HbA1c, and insulin among participants without type 2 diabetes.

Lymphocyte subpopulation	ln-Glucose (*n* = 795)		HbA1c (%) (*n* = 623)		ln-Insulin (*n* = 552)	
	B (SE)	P-value	B (SE)	P-value	B (SE)	P-value
**%Natural Killer** (per 5%)						
Model 1	0.009 (0.005)	0.08	0.01 (0.02)	0.57	0.02 (0.03)	0.46
Model 2	0.007 (0.005)	0.14	0.008 (0.02)	0.65	0.004 (0.02)	0.88
**%γδ T** (per 0.86)						
Model 1	0.005 (0.005)	0.31	-0.01 (0.02)	0.41	-0.002 (0.03)	0.95
Model 2	0.005 (0.005)	0.33	-0.02 (0.02)	0.33	-0.004 (0.02)	0.86
**%Naive** (per 14%)						
Model 1	-0.02 (0.005)	0.0008	-0.002 (0.02)	0.91	-0.09 (0.03)	0.002
Model 2	-0.01 (0.005)	0.01	0.008 (0.02)	0.65	-0.05 (0.02)	0.04
**%Memory** (per 15%)						
Model 1	0.01 (0.005)	0.02	-0.007 (0.02)	0.66	0.04 (0.03)	0.12
Model 2	0.009 (0.005)	0.06	-0.01 (0.02)	0.45	0.03 (0.02)	0.21
**%Th1** (per 8%)						
Model 1	0.006 (0.005)	0.21	0.005 (0.02)	0.75	0.05 (0.03)	0.09
Model 2	0.004 (0.005)	0.46	0.001 (0.02)	0.99	0.02 (0.02)	0.32
**%Th2** (per 0.7%)						
Model 1	0.002 (0.005)	0.72	-0.02 (0.02)	0.39	0.02 (0.03)	0.56
Model 2	0.001 (0.005)	0.91	-0.02 (0.02)	0.33	0.003 (0.03)	0.91

Analyses excluded participants with pharmacologically-treated type 2 diabetes. Lymphocyte subpopulations were analyzed in separate models per SD increment higher. Model 1: Age, gender, race/ethnicity. Model 2: Age, gender, race/ethnicity, and BMI.

%Naive was significantly associated with fasting insulin (*n* = 552) (Table 3). Each SD higher %naive was associated with a 0.09 lower lninsulin (14% of a SD in lninsulin). The results were partially attenuated after additional adjustment for BMI, but remained statistical significant (Table 3). Adjusting for waist circumference in place of BMI did not alter the results. There were no statistically significant associations of %NK, %γδ T, %Th1, or %Th2 with fasting glucose or insulin, or among any of the lymphocyte subpopulations with HbA1c (*n* = 623) (Table 3).

For all results, sensitivity analyses were performed that included additional adjustment for hypertension (*n* = 457) or statin use (*n* = 266), that excluded participants using oral or inhaled steroids (*n* = 42), or that excluded participants with prevalent CVD at exam 4 (*n* = 53), and were not substantially altered.

## Discussion

We have demonstrated that a higher degree of chronic adaptive immune activation, reflected by higher memory and lower naive CD4^+^ T cell proportions, was associated with prevalent type 2 diabetes in a population-based multi-ethnic cohort. There were no associations of NK, γδ T, Th1 or Th2 lymphocyte subpopulations with diabetes, glucose, HbA1c, or insulin.

Our results identifying associations of higher memory and lower naive CD4^+^ T cells with type 2 diabetes is consistent with a role of chronic adaptive immune activation and exhaustion augmenting inflammation during the pathogenesis of type 2 diabetes. However, in this cross-sectional analysis we cannot ascertain the directionality of the relationships, and longitudinal studies evaluating the prospective relationships of biomarkers of chronic adaptive immune activation and immune senescence with incident diabetes will be important.

Relationships of higher memory and lower naive CD4^+^ T cells with type 2 diabetes may reflect both direct and/or indirect mechanisms. T cell activation by diabetogenic or obesity-associated antigens [36] could directly link T-cell responses to type 2 diabetes etiology, consistent with estimates that ~10% of adults with type 2 diabetes have antibodies targeting pancreatic islet cells [37]. Pathogens with cross-reactivity (i.e., antigenic mimicry) to beta cell antigens, such as coxsackievirus B1, have been implicated in autoimmune T-cell activation in type 1 diabetes and may have implications for T-cell activation in type 2 diabetes [38].

Activation of CD4^+^ memory cells that recognized non-pancreatic beta cell autoantigens, such as e.g., oxidized LDL or intestinal-derived products [39], by a proinflammatory cytokine environment generated during obesity or type 2 diabetes may also account for these relationships. In our study, adjusting for measures of adiposity partially diminished the associations of %memory and %naive cells with type 2 diabetes. These findings are consistent with a role of T-cell infiltration, activation, and inflammation during adipose tissue expansion and impaired glucose homeostasis [12, 18, 21, 22, 40, 41].

Alternatively, chronic adaptive immune activation (from e.g., CMV) and subsequent loss of function, such as occurs with age-associated immune senescence [42], may play an indirect role in type 2 diabetes by transferring the responsibility of the immune response onto innate immunity (inflammation) as the adaptive immune system loses functionality [43, 44]. Consistent with this hypothesis, adjusting for CMV and *H*. *pylori*, correlates of CD4^+^ memory and naive cell subpopulations in MESA [33], reduced the associations of %memory with type 2 diabetes. Our previous demonstration of associations of high memory and low naive cells with IL-6 and subclinical atherosclerosis [33] implicates chronic adaptive immune activation and loss as an important mechanism in other inflammatory diseases [45].

Memory and naive cells were not associated with IFG. These null relationships may indicate the importance of an inflammatory or metabolic threshold required for T-cell activation that is surpassed in the later stages of disease and may suggest adaptive immune activation plays a more important role in the progression from prediabetes to diabetes than in the progression of normal glucose status to prediabetes. Previous studies have demonstrated an increased proinflammatory state among individuals with prediabetes who are predominantly insulin resistant, but not among those with primary defects in β-cell function and insulin secretion [46]. We did not have measures of insulin sensitivity or secretion and cannot rule out the importance of chronic adaptive immune activation among individuals with prediabetes characterized by insulin resistance.

Our cross-sectional results did not implicate NK, γδ T, Th1, or Th2 cells in type 2 diabetes. Imbalances of higher Th1, Th17, Th22 and lower Treg ratios have, however, been reported in the peripheral blood of subjects at risk for [22, 26], and with, type 2 diabetes [23–25]. There is also some evidence that insulin treatment may promote a bias towards a Th2 phenotype and impair Treg functionality *in vitro* [47, 48]. Discrepancies with previous studies may be explained by differences in study design. Our epidemiological study cohort was comprised of a free-living population with enrollment criteria determined by clinical CVD and not diabetes status. As such, our study population was not selected based on diabetes prevalence or risk and did not directly compare participants with diabetes to those with normal fasting glucose matched for other characteristics. Our findings may not be generalizable to cohorts with prevalent clinical CVD. Further, as some participants had prevalent type 2 diabetes prior to cellular phenotyping during MESA exam 4, and because we measured lymphocyte populations in peripheral blood, our null results may not reflect the involvement of lymphocyte subsets localized to specific tissues, such as adipose tissue or skeletal muscle, during the initiation of IR or progression to diabetes.

As Th17- and Th22-dependent inflammation are implicated in type 2 diabetes [23–25, 27, 49, 50], and do not directly correlate with Th1 cell responses [51], it is expected that our phenotyping assays missed the T-cell subpopulations responsible for the associations of memory and naive cells with type 2 diabetes. Decreased expression of NK cell-activating receptors and impaired NK function were recently reported among type 2 diabetes patients [28], however we did not evaluate NK receptor expression in our study. Discrepant results between our and other studies may also be explained by smaller sample sizes among previous clinical or pilot studies, demographic differences, or the cross-sectional analyses or limited statistical power in our study.

Several additional limitations are also acknowledged. First, the study is cross-sectional in design and residual confounding and reverse causality are possible. Second, we were limited to using fasting glucose measures to assess glycemic status. Since an oral glucose tolerance test (OGTT) was not performed, it is possible participants with diabetes were misclassified as normal or impaired fasting glucose status. Misclassification would be expected to bias our results toward the null. Therefore, our findings are likely conservative, and this potential misclassification may partially explain null findings observed for the other lymphocyte subpopulations, such as Th1. The sample sizes in each of the glucose categories were small limiting our statistical power to detect differences (as described in Methods). We also did not have information allowing for the classification of latent autoimmune diabetes of adults. Third, insulin and HbA1c were measured during MESA exam 5 among fewer participants which may also bias our results towards the null. Finally, since appropriate methods were not available at the time the MESA-Inflammation protocol was developed, we did not evaluate Th17, Th22, or Treg subpopulations. Specific markers of immune senescence, such as CD28 deficiency, were also not evaluated.

Strengths of the study include the demographic diversity, distribution of obesity status, predominantly CVD-free health status of the study population, and the comprehensive quality assurance / quality control program implemented for the cellular phenotyping measures [32, 33].

In summary, higher memory and lower naive CD4^+^ T cells were associated with type 2 diabetes in a multi-ethnic population-based cohort. These results are consistent with the hypothesis that chronic adaptive immune activation and immune senescence enhance chronic inflammatory diseases by placing a greater burden on the innate immune system. These findings suggest the importance of prospective studies evaluating adaptive immune activation and immune senescence during the progression of type 2 diabetes.

## Supporting Information

S1 Fig Linear regression analyses of natural killer and γδ T cell measurements in fresh whole blood versus 24-hour post-draw samples. The X-axis represents values from freshly processed whole blood and the Y-axis represents values from whole blood processed 24-hours post-draw (*n* = 15). (A) %natural killer (NK) cells; (B) %γδ T cells. NK and γδ T cell subpopulations were expressed as a percentage of lymphocytes.
(TIF)

S2 Fig Distributions of natural killer and γδ T cells in MESA-Inflammation. Distributions of (A) natural killer and (B) γδ T cells are shown in the overall study population. X-axis: Cell value expressed as a percentage of lymphocytes; Y-axis: Frequency observed in the study population.
(TIF)

S3 Fig Typical flow cytometric data for γδ T cell and natural killer (NK) cell subpopulations. (A) Lymphocytes (≥30,000) from whole blood were gated based on their forward (FCS; Y-axis) and side scatter (SCS; X-axis). (B) γδ T cells were identified by positive surface staining for CD3 (X-axis) and γδ T cell receptor (TCR) (Y-axis) (CD3^+^γδTCR^+^). Natural killer lymphocyte populations were gated by negative surface staining for CD3 (C) and identified by positive surface staining for CD56 (Y-axis) and CD16 (X-axis) (CD3^-^CD56^+^CD16^+^).
(TIF)

S4 Fig Typical flow cytometric data for lymphocyte, CD4^+^ memory, CD4^+^ naive, Th1, and Th2 cell populations. Lymphocytes (≥30,000) from whole blood (A) and peripheral blood mononuclear cells (D) were gated based on their forward (FCS; Y-axis) and side scatter (SCS; X-axis). T helper lymphocyte populations were gated by positive surface staining for CD4 (Y-axis, panels B, C, E, and F). CD4^+^ memory cells were identified by positive surface staining for CD45RO (B) and CD4^+^ naive cells were identified by positive surface staining for CD45RA (C). Th1 cells were gated by positive intracellular staining for interferon-gamma (IFN-γ) (E). Th2 cells were gated by positive intracellular staining for interleukin-4 (IL-4) (F).
(TIF)

S1 File Supplemental Materials and Methods. (DOCX)
