# Supplementary figures and images for: Associations of Circulating Lymphocyte Subpopulations with Type 2 Diabetes: Cross-Sectional Results from the Multi-Ethnic Study of Atherosclerosis (MESA)
Source: PLoS One. 2015 Oct 12;10(10):e0139962. doi: 10.1371/journal.pone.0139962 (PMC4601795; doi:10.1371/journal.pone.0139962)

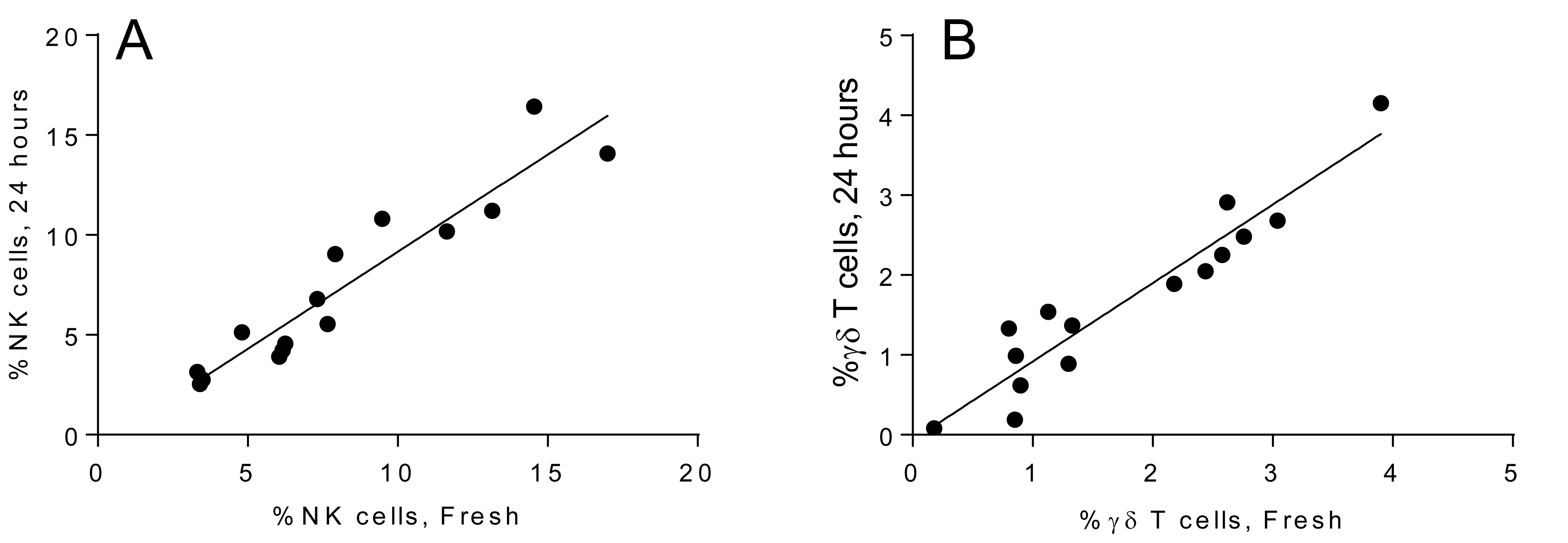

Supplement: S1 Fig — The X-axis represents values from freshly processed whole blood and the Y-axis represents values from whole blood processed 24-hours post-draw (n = 15). (A) %natural killer (NK) cells; (B) %γδ T cells. NK and γδ T cell subpopulations were expressed as a percentage of lymphocytes. (TIF) [file pone.0139962.s001.tif]

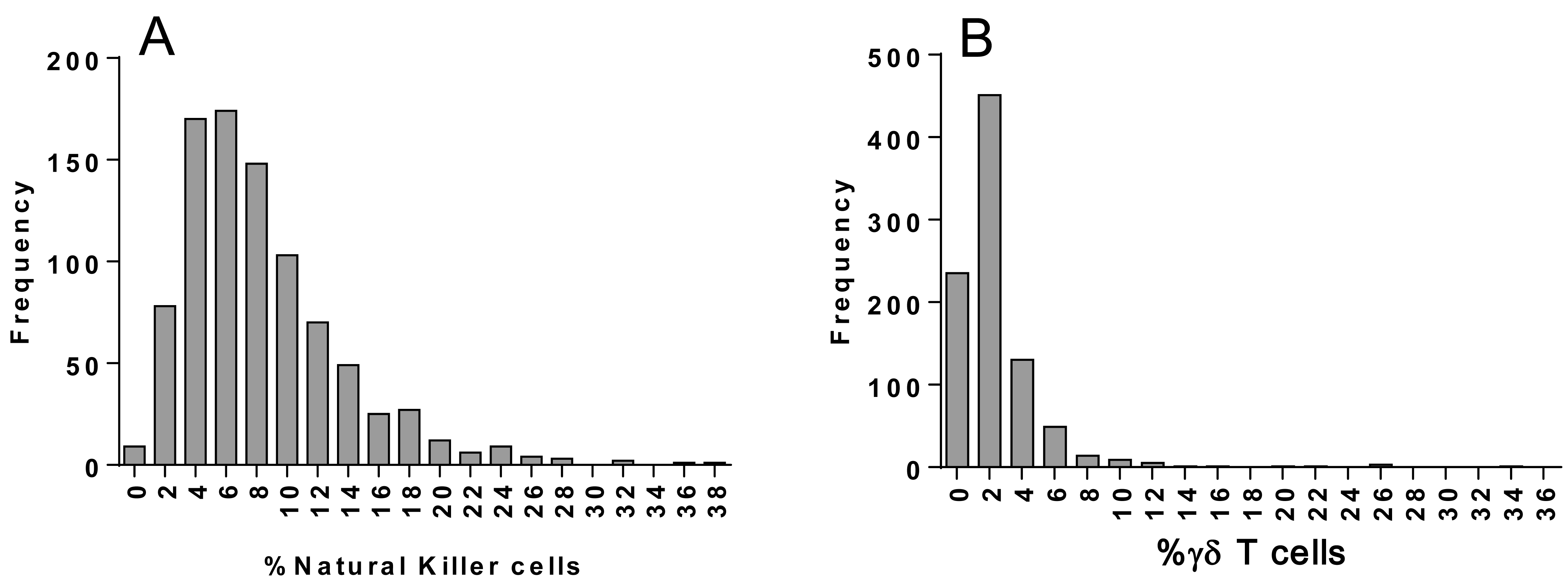

Supplement: S2 Fig — Distributions of (A) natural killer and (B) γδ T cells are shown in the overall study population. X-axis: Cell value expressed as a percentage of lymphocytes; Y-axis: Frequency observed in the study population. (TIF) [file pone.0139962.s002.tif]

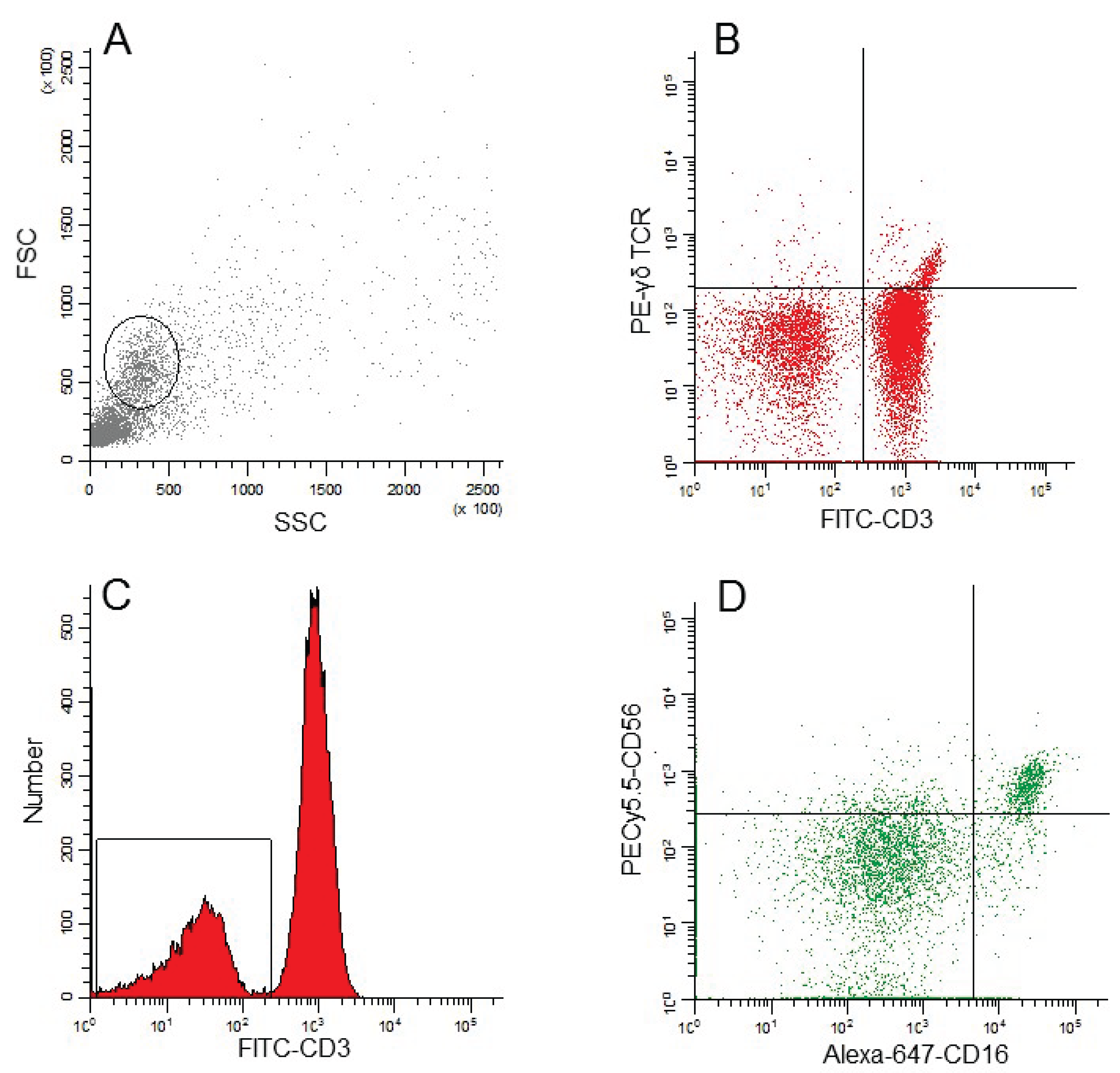

Supplement: S3 Fig — (A) Lymphocytes (≥30,000) from whole blood were gated based on their forward (FCS; Y-axis) and side scatter (SCS; X-axis). (B) γδ T cells were identified by positive surface staining for CD3 (X-axis) and γδ T cell receptor (TCR) (Y-axis) (CD3+γδTCR+). Natural killer lymphocyte populations were gated by negative surface staining for CD3 (C) and identified by positive surface staining for CD56 (Y-axis) and CD16 (X-axis) (CD3-CD56+CD16+). (TIF) [file pone.0139962.s003.tif]

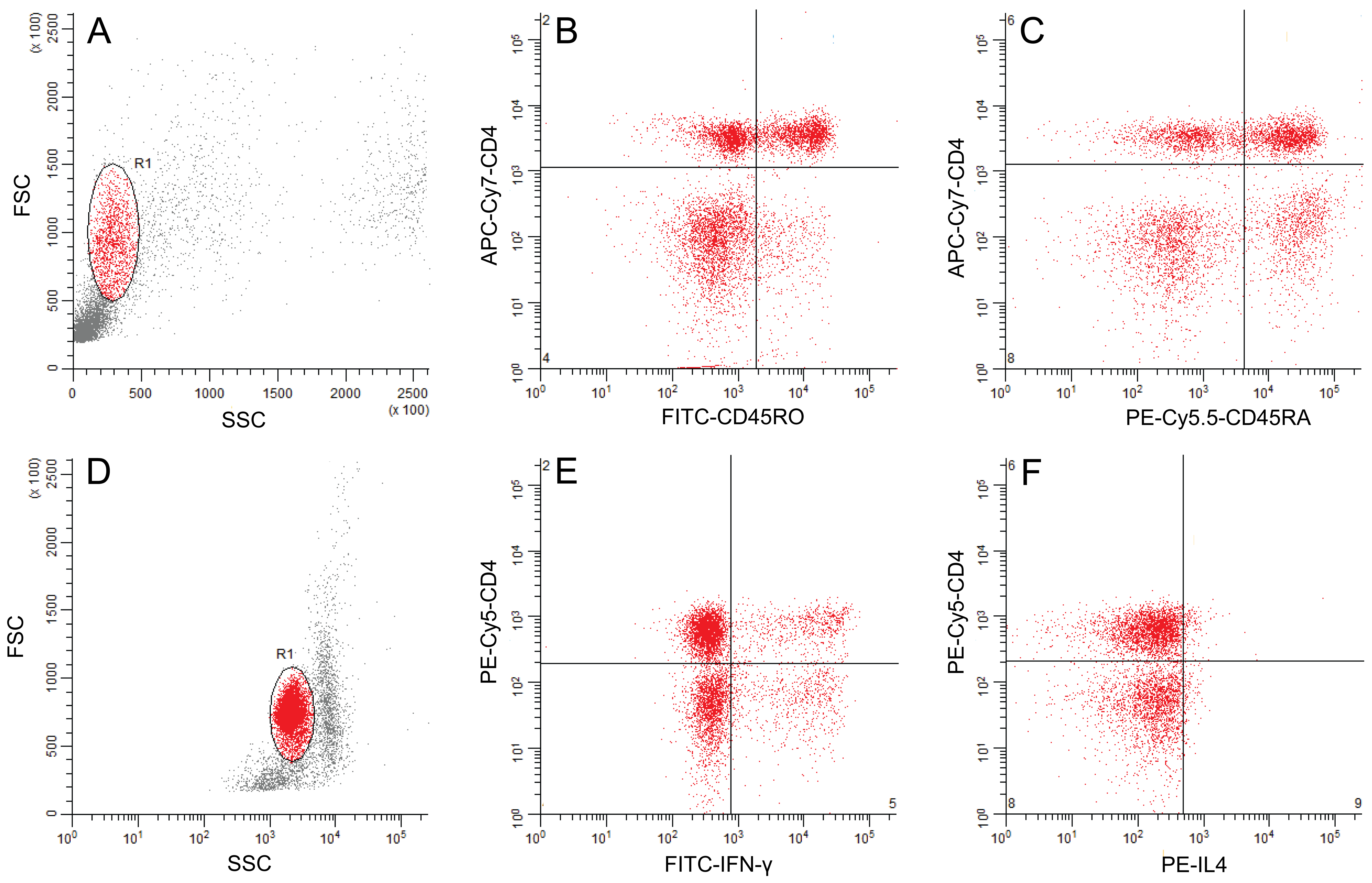

Supplement: S4 Fig — Lymphocytes (≥30,000) from whole blood (A) and peripheral blood mononuclear cells (D) were gated based on their forward (FCS; Y-axis) and side scatter (SCS; X-axis). T helper lymphocyte populations were gated by positive surface staining for CD4 (Y-axis, panels B, C, E, and F). CD4+ memory cells were identified by positive surface staining for CD45RO (B) and CD4+ naive cells were identified by positive surface staining for CD45RA (C). Th1 cells were gated by positive intracellular staining for interferon-gamma (IFN-γ) (E). Th2 cells were gated by positive intracellular staining for interleukin-4 (IL-4) (F). (TIF) [file pone.0139962.s004.tif]
