# Supplementary material for: Associations of Circulating Lymphocyte Subpopulations with Type 2 Diabetes: Cross-Sectional Results from the Multi-Ethnic Study of Atherosclerosis (MESA)
Source: PLoS One. 2015 Oct 12;10(10):e0139962. doi: 10.1371/journal.pone.0139962 (PMC4601795; doi:10.1371/journal.pone.0139962)
Supplement: S1 File — (DOCX) [file pone.0139962.s005.docx]

**Supporting Information**

Associations of circulating lymphocyte subpopulations with type 2 diabetes: cross-sectional results from the Multi-Ethnic Study of Atherosclerosis (MESA)

Nels C. Olson^1^, Margaret F. Doyle^1^, Ian H. de Boer^2,3^, Sally A. Huber^1^, Nancy Swords Jenny^1^, Richard A. Kronmal^4^, Bruce M. Psaty^5,6^, and Russell P. Tracy^1, 7*^

Departments of Pathology and Laboratory Medicine^1^ and Biochemistry^7^, University of Vermont College of Medicine, Burlington, Vermont, United States of America

^2^Division of Nephrology, Department of Medicine, and ^3^Kidney Research Institute, University of Washington, Seattle, Washington, United States of America

^4^Collaborative Health Studies Coordinating Center, Department of Biostatistics, University of Washington, Seattle, Washington, United States of America

^5^Departments of Medicine, Epidemiology, Health Services, and Cardiovascular Health Research Unit, University of Washington, Seattle, Washington, United States of America

^6^Group Health Research Institute, Group Health Cooperative, Seattle, Washington, United States of America

* Corresponding author

**Cellular phenotyping quality assurance measures**

Protocol development and quality assurance measures for the MESA-Inflammation study have been reported [1, 2]. During MESA exam 4 (2005-2007) heparinized blood was collected at the 6 MESA field centers. Samples were shipped overnight to the Laboratory of Clinical Biochemistry Research at the University of Vermont in specialized containers that maintained a temperature of 15-30°C and were processed for cellular phenotyping upon receipt.

Cellular phenotypes were tested for overnight stability in a group of 13-20 apparently healthy local volunteers. Cell phenotypes were measured from samples of freshly drawn blood and compared with blood samples that were placed in shipping containers, subjected to standard shipping procedures, and measured 24-hours post-draw. Results from these studies for CD4^+^ naive, CD4^+^ memory, Th1, and Th2 phenotypes have been reported [1, 2].

As shown in S1 Fig., the regression slope comparing fresh versus 24-hour post-draw phenotypes for natural killer (NK) cells was 0.97; the Pearson correlation coefficient was 0.94. The regression slope for γδ T cells was 0.98 and the Pearson correlation coefficient was 0.95. These results indicated the measurements for NK and γδ T cells were stable over the two time points.

We assessed biovariability and assay protocol drift throughout the duration of the MESA-Inflammation study by enrolling 14 apparently healthy local volunteers. The volunteers donated samples prior to the beginning of the study and periodically throughout the study’s duration allowing for repeated measures of the cellular phenotypes. These measurements were used to calculate the analytical coefficient of variation (CV) (CVa); the within-subject CV (CVi); and, the between-subject CV (CVg) using a nested analysis of variance [3]. In cases where analysis was done only in singleton, no CVa was calculated. The Index of Individuality (II), which measures the ability to detect changes in an individual compared with the total population, was calculated as the ratio of the within-individual variation (CVi) to the between-subject variation (CVg). The biovariability data for NK and γδ T cells are summarized in S1 Table.

**S1Table.** Biovariability measures for natural killer and γδ T cells.

| **Lymphocyte subpopulation** (*n*=14) | **Mean (SD)** | **% CVi** | **% CVg** | **II** | **CVg / CV_total_** |
| --- | --- | --- | --- | --- | --- |
| % Natural Killer Cells | 6.6 (3.2) | 33.9 | 33.8 | 1.01 | 0.50 |
| % γδ T Cells | 2.0 (1.3) | 40.4 | 56.5 | 0.72 | 0.58 |

CVi, intra-person coefficient of variation; CVg, inter-person coefficient of variation; II, index of individuality (CVi/CVg); CV_total_=CVi+CVg.

**References**

1. Tracy RP, Doyle MF, Olson NC, Huber SA, Jenny NS, Sallam R, et al. T-helper type 1 bias in healthy people is associated with cytomegalovirus serology and atherosclerosis: the multi-ethnic study of atherosclerosis. J Am Heart Assoc. 2013;2(3):e000117.

2. Olson NC, Doyle MF, Jenny NS, Huber SA, Psaty BM, Kronmal RA, et al. Decreased naive and increased memory CD4(+) T cells are associated with subclinical atherosclerosis: the multi-ethnic study of atherosclerosis. PLoS One. 2013;8(8):e71498.

3. Sakkinen PA, Macy EM, Callas PW, Cornell ES, Hayes TE, Kuller LH, et al. Analytical and biologic variability in measures of hemostasis, fibrinolysis, and inflammation: assessment and implications for epidemiology. Am J Epidemiol. 1999;149(3):261-7.
